# Space-Borne GNSS-R Ionospheric Delay Error Elimination by Optimal Spatial Filtering

**DOI:** 10.3390/s20195535

**Published:** 2020-09-27

**Authors:** Qiuyang Zhang, Yang Liu, Junming Xia

**Affiliations:** 1 School of Instrumentation and Opto-Electronic Engineering, Beihang University, Beijing 100191, China; sy1817123@buaa.edu.cn; 2 National Space Science Center, Chinese Academy of Science, Beijing 1001901, China; xiajunming@nssc.ac.cn

**Keywords:** space-borne GNSS-R, sea surface altimetry, ionospheric delay, spatial filtering, reflectometry

## Abstract

Global Navigation Satellite System Reflectometry (GNSS-R) technology is a new and promising remote sensing technology, especially satellite-based GNSS-R remote sensing, which has broad application prospects. In this work, the ionospheric impacts on space-borne GNSS-R sea surface altimetry were investigated. An analysis of optimal values for spatial filtering to remove ionospheric delays in space-borne GNSS-R altimetry was conducted. Considering that there are few satellite-borne GNSS-R orbit observations to date, simulated high-resolution space-borne GNSS-R orbital data were used for a comprehensive global and applicable study. The curves of absolute bias in relation to the bilateral filtering points were verified to achieve the minimum absolute bias. The optimal filtering points were evaluated in both statistical probability density and quantile analysis to show the reliability of the selected values. The proposed studies are helpful and valuable for the future implementation of high-accuracy space-borne GNSS-R sea surface altimetry.

## 1. Introduction

Global Navigation Satellite System Reflectometry (GNSS-R) technology is a promising remote sensing technology, especially satellite-based GNSS-R remote sensing, which has broad application prospects [1,2]. Compared with traditional microwave remote sensing technology, GNSS-R has the advantages of low power consumption, light weight, small size, low cost, and passivity. Since it can receive reflection signals from multiple satellites at the same time, its spatial resolution is higher than that of traditional microwave remote sensing technology, and its data can fill some areas that cannot be covered by traditional remote sensing technology. Currently, GNSS-R has been widely used for the remote sensing of sea surface winds and heights, soil assessments, and ice status monitoring.

GNSS-R sea surface altimetry is currently a research focus. At present, a large number of ground and airborne GNSS-R altimetry experiments have been carried out, which have fully verified the effectiveness of this technology [3,4,5,6]. The development of ground and airborne GNSS-R altimetry will promote the application of this technology in satellite platforms. Compared to traditional sea surface altimetry, this technology can cover a vast region of the sea and help measure the sea surface height from a global view. Space-borne GNSS-R sea surface altimetry uses the single difference between the reflected navigation signal from the sea surface and the direct signal from the navigation satellite simultaneously received by the GNSS-R receiver loaded on a low earth orbit (LEO) satellite to retrieve the sea surface height. For the first time, researchers can use TDS-1 data to retrieve sea surface height experiments [7]. For the two different regions considered, the difference between themean sea surface height (MSSH) of DTU10 [8] is 8.1 and 7.4 m, which is within a tolerable level. The corresponding influence that affects sea surface altimetry accuracy was provided after the performance analysis of TDS-1 data. The altimetry performance was carried out by analyzing the dataset covering the global ocean surface above 60° latitudes in the Northern and Southern Hemispheres. Compared with the MSSH, under an integration time of 1 s, the sea surface height residual is 6.4 m in the 1*σ* condition. Later, the altimetry model was further optimized, and GNSS-R altimetry experiment results using Cyclone Global Navigation Satellite System (CYGNSS) data in the Indonesian Ocean were analyzed; the accuracy was improved to 6 m at the same integration time [9,10]. Another lake water altimetry experiment was carried out with TDS-1 data. The root mean square error between the measured height results and the results provided by the traditional altimeter was relatively large, but there was very high correlation between the two results, with a correlation coefficient of 0.95. These results provided supportive information for further GNSS-R applications in inland water height altimetry [11]. An altimetry experiment with the high incidence angle carrier phase method was realized for the first time by using CYGNSS data. The carrier phase was extracted from the scattering signal. The altimetry accuracy reached 3 cm for median values and 4.1 cm for mean values at a 20 Hz sampling frequency. This result was comparable to the accuracy of traditional altimetry satellites [12].

The accuracy of space-borne GNSS-R sea surface altimetry relies greatly on GNSS line-of-sight and reflecting signal propagation, and the range error of GNSS signals has a great impact on the accuracy of this technology. According to the baseline theory of the GNSS, ranging errors can be induced by satellite ephemerides, clocks, and atmospheric propagation paths, among which the ionosphere serves as a critical source of ranging error [13]. The ionospheric delay also affects the accuracy of space-borne GNSS-R sea surface altimetry since both the line-of-sight signal and the reflecting signal propagate through the ionosphere with different paths, leading to a complicated geometry for error analysis. To address this problem, Camp et al. studied the specific impact of ionospheric scintillation on GNSS-R signals and evaluated the impact of ionospheric scintillation on the signal-to-noise ratio, which is a key factor affecting the accuracy of GNSS-R altimetry [14]. The existing signal that can be used for height measurement is mainly the L1 band frequency; this single frequency cannot eliminate ionospheric delay. To improve the accuracy of altimetry measurements, dual frequency combinations have been suggested to eliminate ionospheric delays. However, the final altimetry accuracy decreases inversely after the elimination of ionospheric delay by dual frequency combinations. The problem is that the ranging error propagation can greatly skew the accuracy of the final result. Based on this fact, other concepts were proposed to eliminate the ionospheric delay. Considering the fact that there is no correlation between the ocean elevation signal and the ionospheric delay, an assumption can be further made that the spatial variability of the ionosphere ranging error is only limited at a small spatial scale. Under this condition, the method of spatial filtering can be used to reduce the ranging error so that the accuracy of altimetry measurements can be further improved [15]. This assumption is enlightening; however, only two cases have been analyzed. Researchers have stressed the necessity of filtering ionospheric delays before using them to correct the ranging errors in the area of satellite altimetry [16,17].

To address this problem, this paper focuses on the analysis of specific optimal spatial filtering values to remove ionospheric delays in space-borne GNSS-R altimetry. Considering that there are few satellite-borne GNSS-R orbit observations to date, this work uses simulated high-resolution space-borne GNSS-R orbital data for a comprehensive global and applicable study, and the simulated orbit characteristics are very similar to those of existing GNSS-R satellites. In Section 2, the baseline theory and method were introduced; in Section 3, the experimental results were presented by the proposed method; and in Section 4, a discussion was conducted. Finally, concluding remarks are presented.

## 2. Materials and Methods

### 2.1. Calculation of Space-Borne GNSS-R Ionospheric Delay

**Figure 1:**
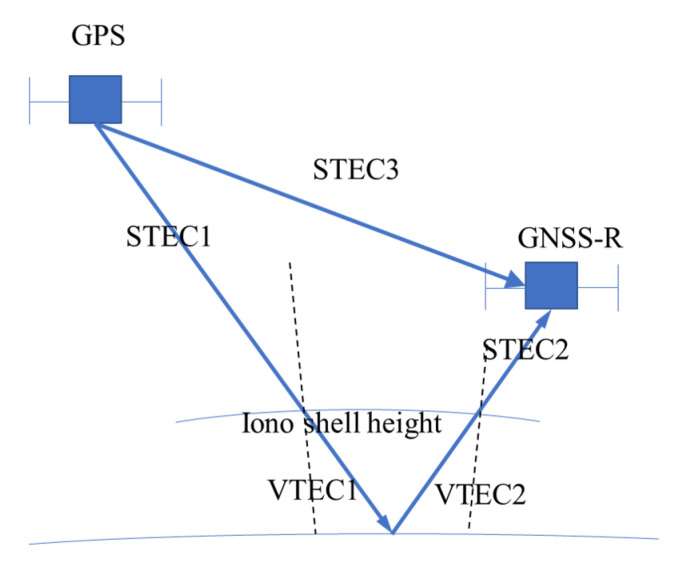
In the GNSS-R ionospheric error model, the ranging error of the ionosphere is estimated for the signals reflected from the Earth using the global ionosphere map (GIM) model with data from International GNSS Service (IGS), and the vertical total electron content (VTEC) value is obtained and then mapped to slant total electron content (STEC) based on different elevations for a single point.

In space-borne GNSS-R altimetry, as shown in Figure 1, the influence of the ionosphere on a GNSS-R signal can be divided into three parts: the line-of-sight ranging error from the trans-ionospheric signal directly from the satellite transmitter to the space-borne receiver in a LEO satellite; the ranging error from the GNSS-R signal satellite transmitter to the mirror reflection point; and the ranging error from the mirror reflection point to the GNSS-R space-borne receiver in the LEO satellite. Among these three paths, the direct path has little effect on the signal delay, so it will not be considered in the ionospheric delay calculations.

Existing GNSS-R satellites, such as TDS-1 and CYGNSS, use only single frequency (L1) measurements. Under such conditions, the ionospheric model is necessary to retrieve ionospheric delays for single frequency measurements. A series of models were proposed and accepted for ionospheric studies. Here, the GIM model is applied in this work to estimate the ionospheric delay for space-borne GNSS-R observations. The GIM is a common model used to study global ionospheric changes and provides global ionosphere total electron content (TEC) [18,19,20]. The global ionosphere has been divided with a longitude step of 5° and a latitude step of 2.5°, making for a total of 5183 grids globally. The GIM is provided by IGS data analysis centres with temporal intervals of 2 hours or even less. The VTEC at the ionospheric pierce points shown in Figure 1 was calculated with the GIM to obtain the VTEC from the GNSS transmitter to the sea surface reflecting point (VTEC1), the sea surface reflecting point to the space-borne GNSS-R receiver (VTEC2) and the STEC from the GNSS transmitter to the space-borne GNSS-R receiver (STEC3). In further calculations, the effect of STEC3 is very minor and can be ignored; thus, only STEC1 and STEC2 were used. For the path of STEC3, the altitude of ionosphere is larger than 600 km, according to the vertical structure of ionosphere, the electron density is mainly concentrated below 450 km in the F2 layer, as revealed by many electron density observations [21]. In high altitude of ionosphere (600–1000 km and above), the impact of ionosphere on GNSS signal is relatively weak.

Once VTEC1 and VTEC2 were calculated, a mapping function was used to project the VTEC to STEC along the signal propagation path, the calculation was represented by Equations (1)–(4) [8]. The mapping function is given as:

(1) $$M_{1,2}=\frac{1}{\sqrt{1-\left(\cos El\cdot \frac{R_{E}}{R_{E}+h}\right)}},$$

where El is the elevation of the satellite at the sea surface reflection point, $R_{E}$ is the radius of the Earth, and h is the height of the ionosphere used in the GIM model. The total ionospheric delay was computed with the combination of STEC1 and STEC2 and is given as:

(2) $$\delta_{1,2}=M_{1,2}\cdot \frac{40.3\times 10^{16}\cdot VTEC_{1,2}}{f_{L1}^{2}}.$$

The total ionospheric delay is finally represented as:

(3) $$\Delta \rho_{IonoMod}=\delta_{1}+\delta_{2}.$$

In the space-borne GNSS-R altimetry model, the height error corresponding to the ionospheric delay is expressed as:

(4) $$\Delta h=\frac{\Delta \rho_{Iono}}{2\cos\theta }\;,$$

where *θ* is the incident angle of the sea surface reflecting point. To use the simulated orbital observation, the ionospheric delay was simulated as:

(5) $$\Delta \rho_{IonoMea}=\Delta \rho_{IonoMod}+\Delta \rho_{IonoNoise},$$

where $\Delta \rho_{IonoNoise}$ is Gaussian noise with standard deviation varying in accordance with the incident angle, which is given as:

(6) $$\sigma_{IonoNoise}=2\sigma_{h}\cdot \cos\theta .$$

Here, supposing $\sigma_{h}=20\;$cm, that is, the influence of ionospheric delay observation noise on height accuracy is 20 cm, then the standard deviation of Gaussian noise will vary with the incident angle of the specular reflection point.

### 2.2. Spatial Filtering of Ionospheric Delay

In space-borne GNSS-R altimetry, the calculation was represented from Equations (7)–(11) [13], the single difference of measurement is given as:

(7) $$\rho =-2h\cos\theta +\frac{I^{\prime }}{f^{2}},$$

where h means the height measured from GNSS-R altimetry. $I^{\prime }$ means the ionospheric delay related to θ, the incidence angle of the single point, f means the frequency of the signal. This formula can be rewritten as:

(8) $$y=-h+\frac{x}{f^{2}},$$

where y=ρ/(2cosθ), $x=I^{\prime }/\left(2cos\theta \right)$.

If the ionospheric delay is corrected by a dual frequency method (assuming L1 and L5 bands are used), the above equation can be expressed as follows at different frequencies:

(9) $$\left[\begin{array}{c} y_{1}\\ y_{2} \end{array}\right]=\left[\begin{array}{cc} -1 & f_{1}^{-2}\\ -1 & f_{5}^{-2} \end{array}\right]\left[\begin{array}{c} h\\ x \end{array}\right].$$

The error propagation function was derived by solving the above equation, which is given as:

(10) $$\frac{\sigma_{h}}{\sigma_{y}}=2.59,\frac{1}{f_{1}^{2}}\sigma_{x}=1.78\sigma_{y},\;\;\frac{1}{f_{5}^{2}}\sigma_{x}=3.2\sigma_{y}.$$

Since the ionospheric delay is spatially related to itself and not related to the topography of the sea, it can be considered that the ionospheric delay changes little (or is smooth) on a limited spatial scale. Therefore, the accuracy of the ionospheric delay can be improved by a regression of multiple continuous sampling points. If the regression is accurate, the accuracy of ionospheric delay estimation will be improved by $\sqrt{n}$ [13], signal n means the number of points to be used for filtering. If the dual frequency method is used to correct the ionospheric delay and spatial filtering is carried out, the final altimetry measurement accuracy can be expressed as follows:

(11) $$\sigma_{h}=\left(\sqrt{\frac{1}{2}+\frac{1.78^{2}}{4n}+\frac{3.20^{2}}{4n}}\right)\sigma_{h,0}.$$

This spatial filtering method is based on two assumptions: (1) the variation of ionospheric delay along an orbit is gentle; (2) since the variation of ionospheric delay is predictable, the accuracy can be improved by effective regression. The proposed spatial filtering method can help reduce the ionospheric delay for space-borne GNSS-R propagating signals, resulting in improvements in altimetry measurement accuracy.

The GNSS-R simulated trajectory data were analyzed by the spatial filtering method in the statistical aspect, the simulated data were provided by the National Space Science Center of China, with over 2 million GNSS-R reflection trajectories in global coverage. The simulated GNSS-R trajectories were quite similar to the real condition, to supplement the vacancy of real space-borne GNSS-R observations for analysis. In the proposed method, the GNSS-R ionospheric delays were derived by Equation (5) with simulated trajectories, then the mean value filter method was used to compare the ratio of MAE (mean absolute error). The optimal filtering performance was decided with the variation curve of filtering points  fn. The experiments were conducted with over 2 million global simulated GNSS-R trajectories to decide the distribution of optimal filtering points. The results were evaluated with two criteria: to find the peak value of fnmin  and to use 99% quantile for  fn. Compared with polynomial regression, the mean value filtering method has good computation efficiency over the polynomial method, and has better accuracy under small and medium spatial scales. 

## 3. Results

### 3.1. Ionospheric Delay from Simulated Orbital Data

**Figure 2:**
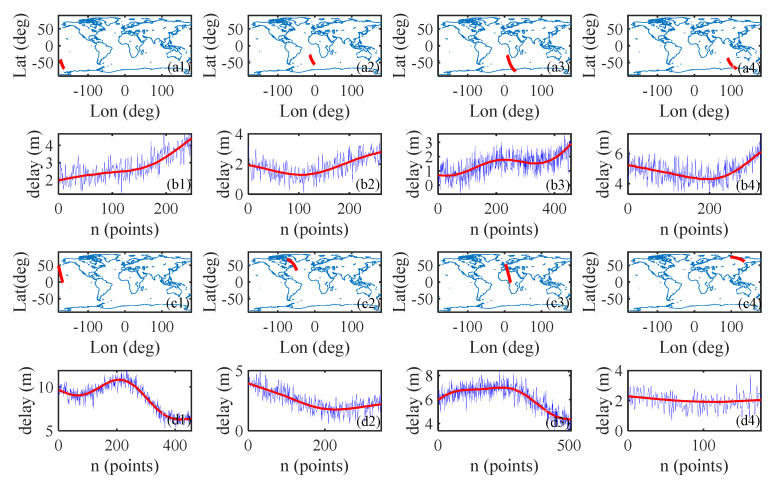
Cases for track, ionospheric delay (red, units m), and observation of ionospheric error (blue, units m) along the track separated from 8 different areas around the earth. Sample n means the sample number along the track, and the sample frequency is 2 second.

Simulated orbital data are used in this experiment, including the Global Positioning System (GPS) satellite trajectory, reflection point trajectory, space-borne receiver trajectory, elevation, azimuth, and incident angle of the reflection point. The ionospheric delay of each sea surface reflection point signal propagating path can be calculated according to the method in Section 2.1. Then, a single frequency ionospheric delay correction of the reflection point trajectory is obtained from the GIM model, and noise is added to the ionospheric delay correction to simulate ionospheric delay measurements. Figure 2 shows the trajectories of reflection points distributed in eight different regions of the world and the corresponding single frequency ionospheric delay measurements. The red curves in Figure 2 show the single frequency ionospheric delay corrections from the GIM, and the blue curves show the ionospheric delay measurements with observation noise.

### 3.2. Spatial Filtering Method Results for Ionospheric Delay

**Figure 3:**
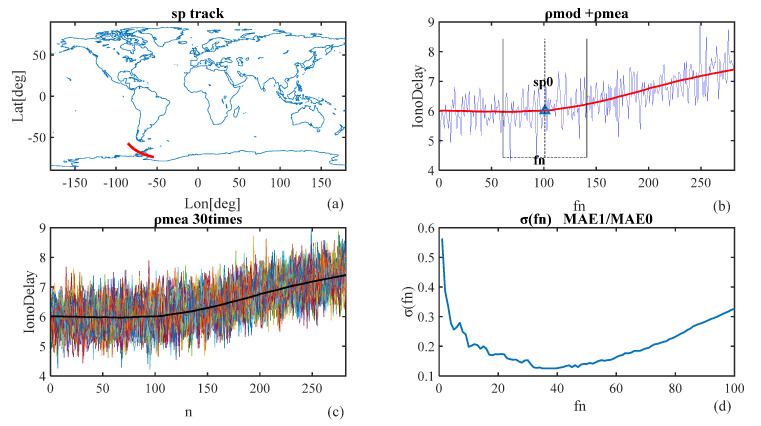
Filtering case: (**a**) track of a single point; (**b**) sample sequence of the observation of ionospheric error and the length of filtering (fn) on both sides of the single point to be estimated by filtering (sp0); (**c**) observation of ionospheric error obtained from modeled value $\rho_{IonoMod}$ adding noise $\rho_{IonoNoise}$ 30 times with the same method to decrease randomity brought by the noise, 30 times were efficient for computation consumption; (**d**) deviation plot (filtering plot) with fn between the estimated and modeled ionospheric error (true value for comparison), with a parameter value σ(MAE/MAE0). MAE and MAE0 indicate the mean absolute error after and before filtering.

The simulated ionospheric delay series for different reflection point trajectories were first analyzed by filtering the mean values. The steps of this analysis were introduced as follows: (1) select a trajectory point named SP0, the point to be estimated or filtered, on a certain reflection trace; (2) take 100 continuous ionospheric delay measurements before and after the point to form an analysis sample with a total of 201 reflection points; (3) take the point SP0 as the center and select fn samples before and after the center separately, where the mean value of these samples is considered the estimation of the center value; fn samples means the number of points before and after the center separately to be used for filtering, called filter n(fn); (4) study the variation of absolute bias in relation to the changes of fn, the absolute bias is the difference between the estimation and the real value derived from the single frequency ionospheric delay correction model. The results are shown in Figure 3. In the results, Figure 3a shows the sample trajectory of SP0; Figure 3b shows the ionospheric delay measurements and correction from the GIM model; Figure 3c shows the variation curve with bilateral filtering distance fn of the deviation σ, the difference between the estimated value of the mean filter and the model value ($\rho_{IonoMod}$); it demonstrates the sequence of 30 dual-frequency ionospheric delay observations for the same orbit trajectory. The observations were derived from model plus noise, containing certain randomity. Figure 3d shows the sequence of 30 ionospheric delay dual frequency observation values for the same orbit trajectory because the observation values are obtained by the modeled value plus noise, so there is certain randomness. Through multiple filtering analysis, the randomness is eliminated; that is, the filtering curve in Figure 3c is the average value of 30 ionospheric delay observation values passing through the same point.

During the mean filtering process, the standard deviation of absolute bias was denoted as *σ* which was computed by the following equation:

(12) $$\sigma \left(fn\right)=\sum_{k=1}^{30}\left(\rho_{ionoEstimated}\left(fn,k\right)-\rho_{ionoMod}\left(fn,k\right)\right),$$

where $\rho_{ionoEstimated}\left(fn\right)$ means the value of ionospheres delay after noise being filtered using fn ( 2fn+1 points around the single point to be analyzed) with the filtering method mentioned above, $\rho_{ionoMod}\left(fn\right)$ is the value of ionospheres delay as a contrast. The step of summing means the average of 30 times estimating.

**Figure 4:**
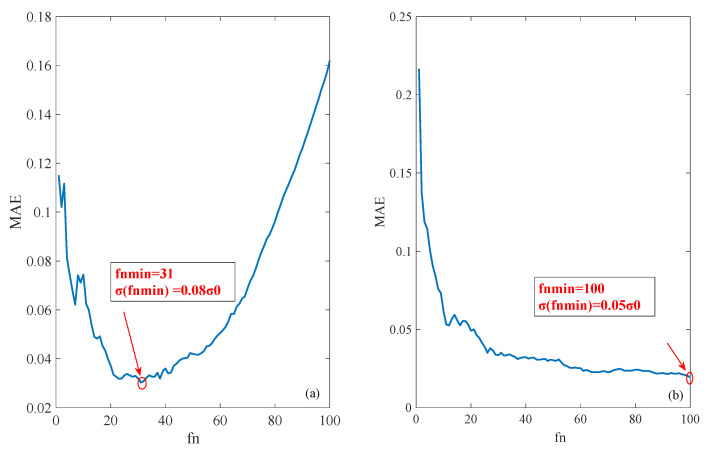
Two classic shapes of the filtering plot.

Figure 4 shows two typical mean filtering curves; with an increment of the sampling points fn, the σ has two variation trends: (a) σ decreases with fast increment of fn; after reaching to a bottom value, σ gradually increases. Thus, an optimal fn can be found to approach the best estimation between the real value and the estimated value, called fnmin. (b) σ decreases drastically with increasing fn and then decreases gradually. Under this condition, an optimal fn can be found to approach the best estimation between the real value and the estimated value. To summarize, an optimal sampling point fnmin can be obtained for ideal filtering.

It can be seen from the above results that the accuracy of ionospheric delay can be greatly improved if fnmin is found after mean filtering. Without loss of generality, a global view of spatial filtering estimation was conducted as follows to obtain the relationship between spatial parameters of longitude, latitude, incident angle, and the optimal fnmin for spatial filtering.

### 3.3. Statistical Analysis of the Spatial Filtering Method

**Figure 5:**
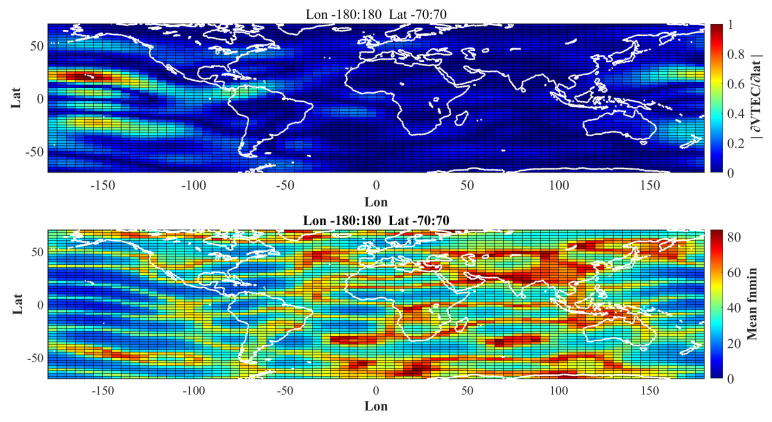
The absolute value of VTEC map derivative with latitude (**top**), and the mean value of fnmin array at each grid (**bottom**). VTEC grid data from IGS, year 2017 doy 1 00:00. GPS constellation data for GNSS-R are used for this analysis.

For this purpose, with the applicable GIM global grids, the reflection points were analyzed from two aspects. First, the global distribution analysis of fnmin was performed by replacing the mean value of fnmin in each grid with the values for all regions in each grid; second, a statistical distribution of fnmin was performed for all the analysed reflection points. Each spatial filtering curve was studied, as shown in Figure 5.

All the reflection points were analysed with the GIM global grids. The average value of fnmin for each grid point replaced all the values in the grid, and the global distribution for the mean value of fnmin was then analyzed. The statistical distribution for the optimal filtering points fnmin and σ(fnmin) of all the reflection points were studied. The statistical distribution for σ(fn) was analyzed when different fn  were considered for analysis.

Figure 5 shows the global distribution of the mean fnmin. Each grid value represents the mean value of all reflection points in the grid. The up plot demonstrates the global VTEC provided by IGS, the VTEC values were differentiated along the latitudinal direction, and the absolute values were considered. The bottom plot shows the global distribution of the mean fnmin. The absolute differentiated VTEC and mean fnmin were negatively correlated, especially in the peak regions of $\left|\frac{\partial \mathrm{VTEC}}{\partial \mathrm{lat}}\right|$.

**Figure 6:**
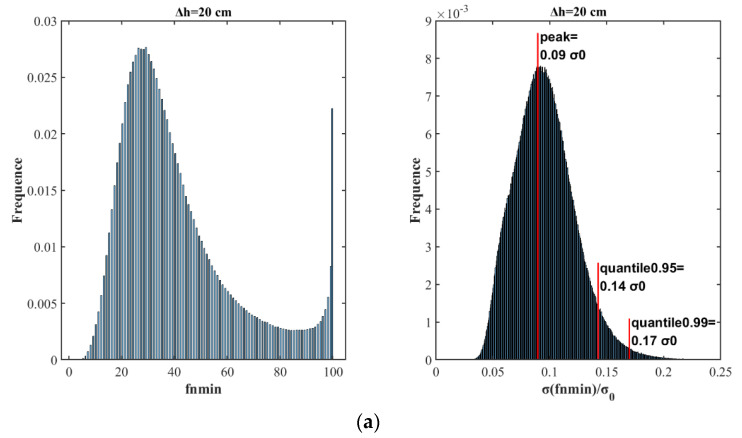

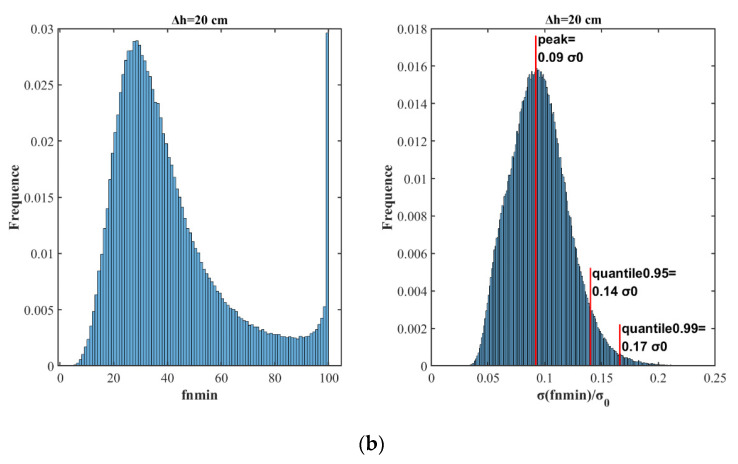
The probability distribution of all samples fnmin and deviation values σ(fnmin). (**a**): GPS-R data; (**b**): BDS-R data.

Each spatial filtering curve was studied, as shown in Figure 6. Most values of fnmin (80%) were concentrated in a range of fn<55, BDS constellation data for GNSS-R fn<55 ;  with peak probability when fn=30 for GPS constellation data for GNSS-R, fn=28 for BDS-R data; and fewer data were distributed in a range between 55 and 100 for both GPS-R and BDS-R data. However, the probability increased drastically when fn=100, this is because the overall filtering length is 201, and fn=100 is the maximum median filtering length. At the same time, the standard deviation of absolute bias decreased greatly with the increment of fn and then decreased gradually, in agreement with Figure 3b. For the statistical analysis of $\sigma_{min}$, it was found that 99% of the $\sigma_{min}$ was less than $0.17\sigma_{0}$, 95% of the $\sigma_{min}$ was less than $0.14\sigma_{0}$, and the peak frequency values of the $\sigma_{min}$ were $0.09\sigma_{0}$.

**Figure 7:**
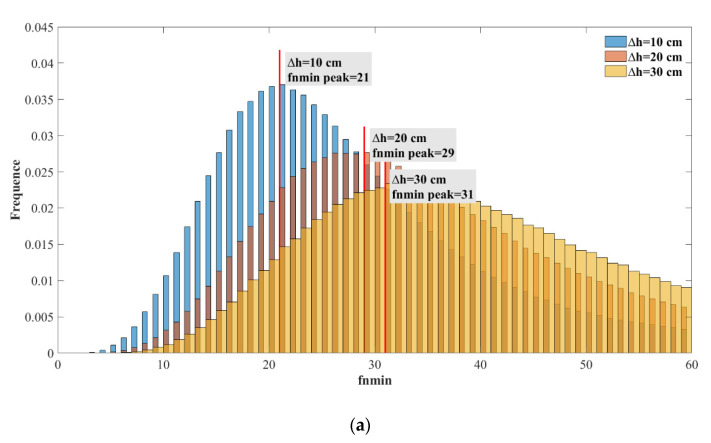

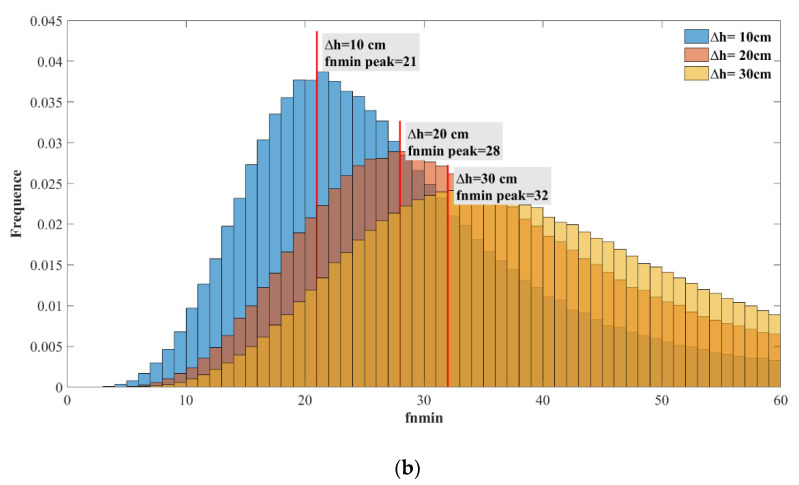
The probability distribution for all samples fnmin when different Gaussian noise was added. GPS-R data are on the top in (**a**) and BDS-R data are on the bottom in (**b**).

**Figure 8:**
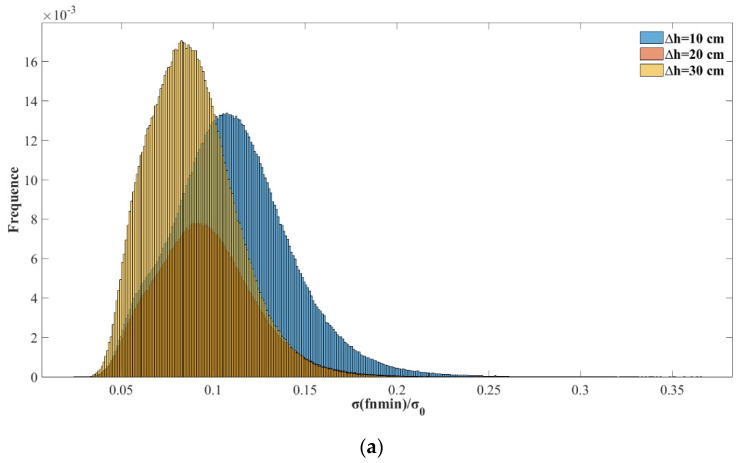

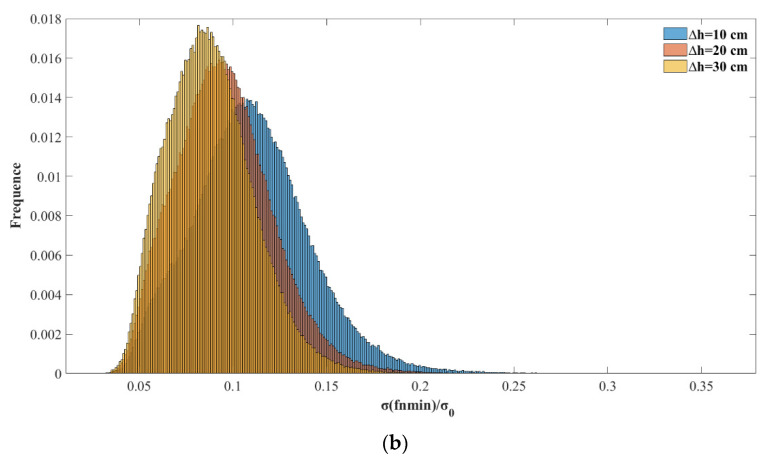
The probability distribution for $\sigma \left(fnmin\right)/\sigma_{0}$ in all samples when different Gaussian noise was added. GPS-R data are on the top in (**a**) and BDS-R data are on the bottom in (**b**).

From the above analysis, it can be concluded that when filtering ionospheric delay observations, there is an optimal fnmin for each reflection point that provides the best filtering. However, fnmin is a random variable and cannot be predicted through the relevant parameters of reflection points. To achieve a better filtering effect, the peak value of fnmin should be selected. When Δh=20 cm, fnmin=29; under such conditions, a greater probability can be obtained to obtain a better filtering effect. Here, if all ionospheric delay samples are filtered with fnmin=29 on both sides, the results are shown in Figure 7. Moreover, for Δh=10, 20, 30 cm, the fnmin distributions were demonstrated, and the corresponding peak values of fnmin were 21, 29, and 31. Figure 7 shows the statistical probability distribution of σ(fnmin) with different Δh values. Each σ(fnmin) value is divided by 2Δhcosθ to compare the change in noise accuracy before and after filtering, and 2Δhcosθ represents the noise accuracy before filtering. Figure 8 shows the distribution $\sigma \left(fnmin\right)/\sigma_{0}$ when Δh=10, 20, 30 cm.

**Figure 9:**
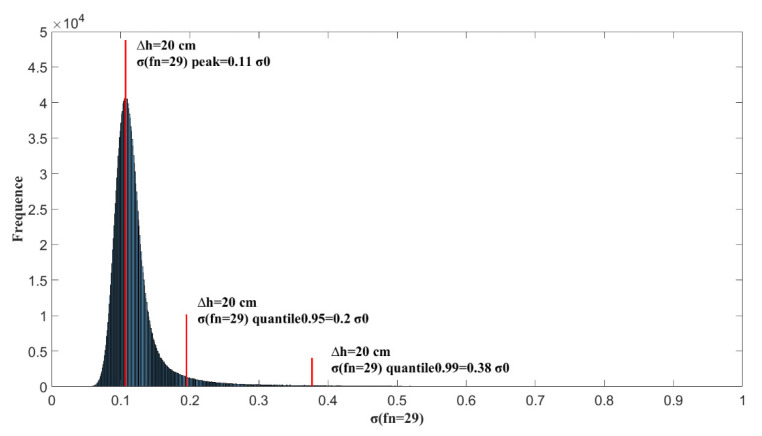
The probability distribution for $\sigma \left(fn=29\right)/\sigma_{0}$ in all samples when the noise accuracy is Δh=20 cm and fn=29 for all samples to be filtered.

If the peak probability for the fnmin distribution is selected during filtering, that is, all points adopt bilateral filtering distance fnmin=29, then the distribution for all filtered $\sigma \left(fn=29\right)/\sigma_{0}$ is shown in Figure 9. Here, the peak values of the probability distribution are 0.1 $\sigma_{0}$, 0.95 quantile 0.21 $\sigma_{0}$, and 0.99 quantile 0.43 $\sigma_{0}$. The 0.95 and 0.99 quantiles can be compared with the values of 2σ and 3σ in the Gaussian distribution. It was found that when the peak value of fnmin=29 is selected to filter the ionospheric delay observations for all reflection points, the filtering effect is not ideal because the peak for $\sigma \left(fn=29\right)/\sigma_{0}$ is nearly 3 times different from that for the 0.99 quantile, and the former is 0.1 $\sigma_{0}$; the latter is 0.4 $\sigma_{0}$. That is, although the filtering result falling within 0.1 $\sigma_{0}$ is the largest, the filtering result is greater than 0.2 $\sigma_{0}$ in nearly 5% of cases, and the filtering result is greater than 0.4 $\sigma_{0}$ in 1% of cases. The fluctuation range of the filtering effect is large. Therefore, according to the method of directly taking fnmin, fn=15~40 can be selected to conduct filtering analysis on all reflection points and observe the distribution of $\sigma \left(fn\right)/\sigma_{0}$ when  fn  takes different values to search for a more stable value of fn.

**Figure 10:**
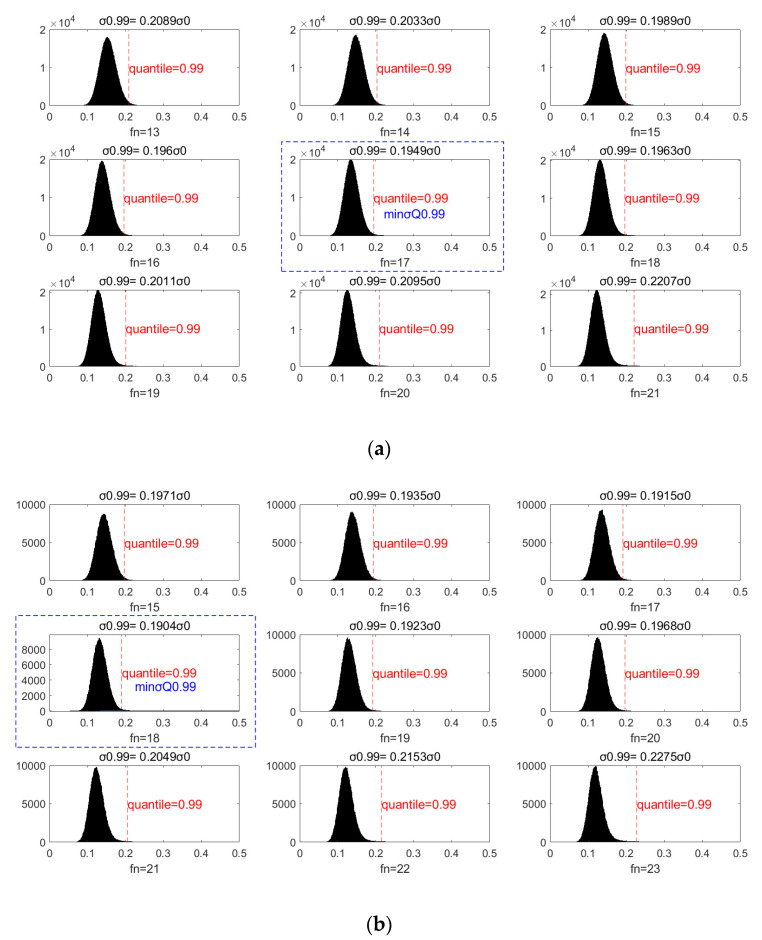
The probability distribution for $\sigma \left(fn=29\right)/\sigma_{0}$ in all samples when the noise accuracy is Δh=20 cm and fn=13~21 for all samples to be filtered. The 0.99 quantile of $\sigma \left(fn\right)/\sigma_{0}$ (σQ0.99) with different fn  were calculated, and the minimum value minσQ0.99 was tagged with a blue box. GPS-R data are on the top in (**a**) and BDS-R data are on the bottom in (**b**).

**Figure 11:**
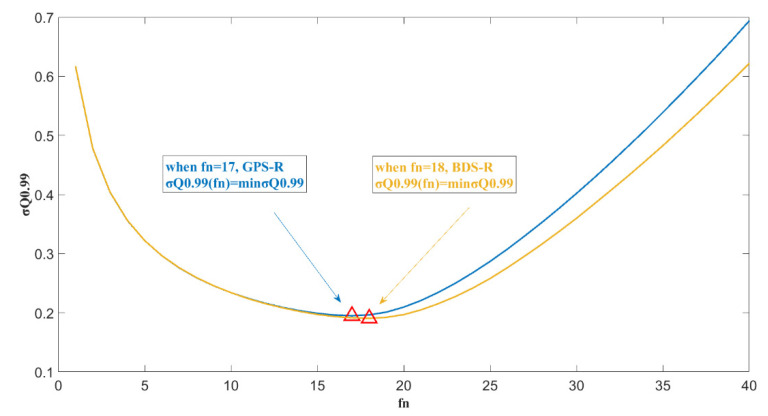
The plot of the σQ0.99 value changing with different fn values,  Δh=20 cm.

Figure 10 shows the probability distribution for $\sigma \left(fn=29\right)/\sigma_{0}$ when the accuracy is 20 cm, and fn is selected for different values in a range from 13 to 21. It can be seen that the 0.99 quantile of $\sigma \left(fn=29\right)/\sigma_{0}$ varies when fn takes different values. That is, under the confidence level of 99%, the accuracy improvement after filtering is different. When fn=17, the 99% quantile of $\sigma \left(fn=29\right)/\sigma_{0}$ reaches a minimum value of 0.195, corresponding to an accuracy improvement of 0.195. Figure 11 shows the 99% quantile variation curve in relation to different fn values. This result shows that this sampling point selection method is more stable.

In this subsection, two methods of filtering point selection were discussed to obtain the optimal filtering. One is to select the point with the highest probability of fnmin  (fnmin peak), which creates a greater probability of obtaining the optimal filtering; the other is to select the appropriate fn to get a better 99% quantile of $\sigma \left(fn\right)/\sigma_{0}$ , which makes the filtering effect more robust.

**Figure 12:**
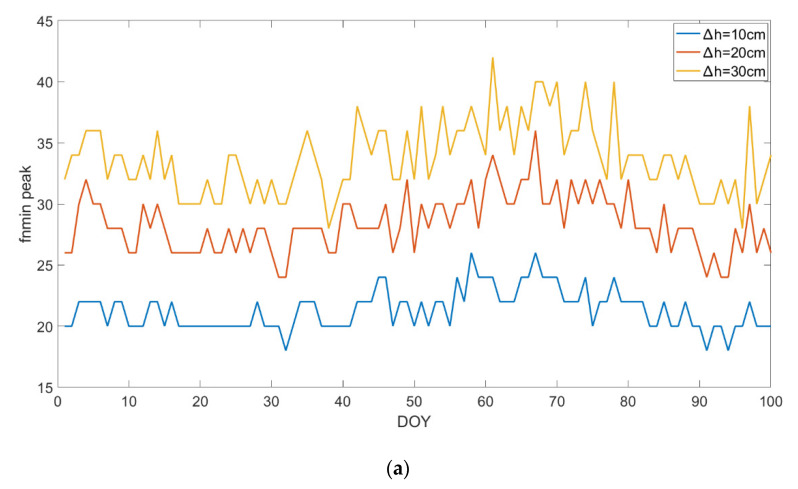

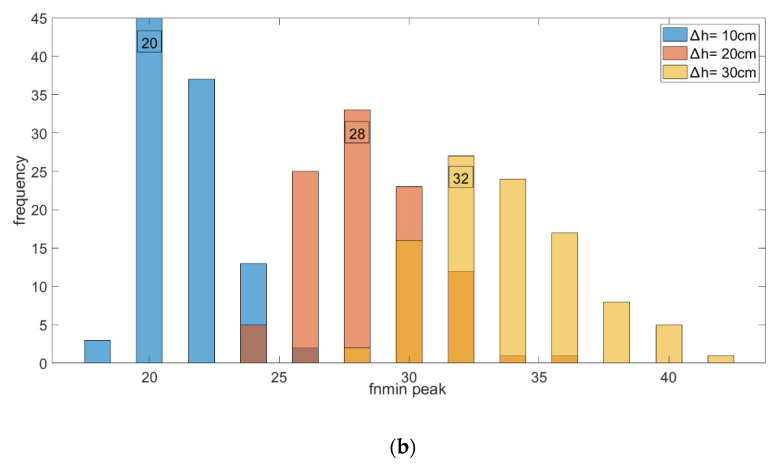
The plot of the fnmin peak value variations with different day (**a**), and the fnmin peak value variations statistical histogram (**b**).

**Figure 13:**
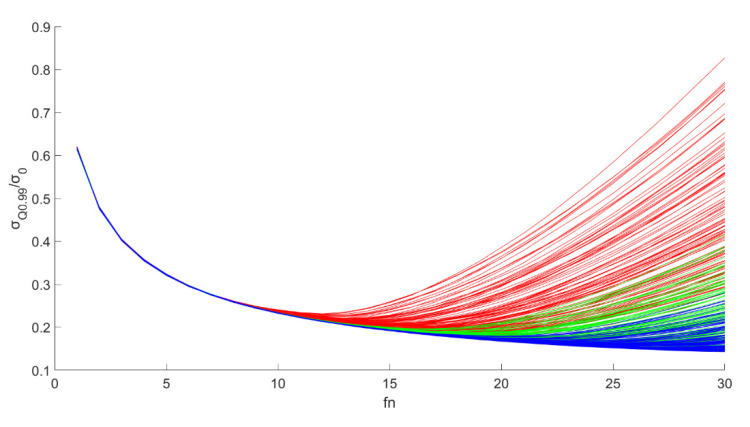
The plots of the σQ0.99 (0.99 quantile of σ) value changing with different fn values, Δh=10, 20, and 30 cm. Red, green, and blue color on behalf of  Δh=10, 20, and 30 cm respectively. Each curve represents one epoch of ionosphere GIM product provided by IGS, with 2-hour interval. One hundred samples were concluded in each plot color.

A large number of epochs of the GIM data were used for modeling the ionosphere delay, the data were selected with 300 days span in 2017. The spatial mean value filtering method was applied with two optimal filtering criteria suggested above, results were demonstrated in Figure 12 and Figure 13. Figure 12a shows fnmin peak value is almost stable for the different time span with minor fluctuation. The histogram of fnmin peak value is described in Figure 12b. The optional value of the filtering point is fnmin peak=20,28, and 32  corresponding to Δh=10, 20, and 30 cm. 

In Figure 13, it is found that the value of $fn_{minQ0.99}$ varies with different epoch, indicating that this method is unstable in temporal domain. However, when the filtering point was limited less than 10, and Δh=10, 20, and 30 cm, the values of $\sigma \left(fn\right)/\sigma_{0}$ were almost the same. fn=10 was used ass the optional value to get a better 99% quantile of $\sigma \left(fn\right)/\sigma_{0}$. Under such condition, the valve of $\sigma_{Q0.99}\;$ was same with different ionospheric epoch, indicating that the distribution of σ(fn=10) was almost same; the value of $\sigma_{Q0.99}$ decreased greatly when fn<10. In the result, when fn=10, $\sigma_{Q0.99}=0.3$, it means that the error after filtering has 99% probability less than 0.3 times of the error before filtering. 

Two methods of filtering point selection are discussed and optimized to obtain the optimal filtering effect with enough validity. The optional filtering point with method are 20,28, and 32 (about 459 km, 638.2 km, and 706 km along the track of single point, the sample frequency is 2 second along the track. In addition, fn is bilateral) corresponding to Δh=10, 20, and 30 cm. The elective filtering point with method is 10 (about 236 km along the SP track) corresponding to Δh=10, 20, and 30 cm.

## 4. Discussion

In the global distribution of fnmin, an obvious correlation was noticed between the mean grid value of fnmin and the absolute value of the zonal differential of the vertical total electron content in this grid. This is because most GNSS-R satellites are inclined orbit satellites, which leads to a single orbit on the earth reference ellipsoid surface. The variation in VTEC along latitude directly determines the change in ionospheric delay in the latitude direction. The correlation coefficient between the mean fnmin and the absolute zonal differential of VTEC was computed to be 0.38, which is not as large as expected. The probable explanation is that fnmin was determined by many factors that cannot be determined only by the zonal differential of VTEC. A better method should be applied to explore the inner mechanism for the correlation between fnmin and the zonal differential of VTEC.

For the statistical distribution of optimal fnmin, the Δh=20cm noise accuracy was set as the background. The statistical distribution of fnmin resembles the Gaussian distribution. Though the GNSS ranging error is timely correlated mainly due to atmospheric effects and multipath, as denoted by [22], it is still reasonable to make Gaussian noise assumption for the ionospheric delay in space-borne GNSS-R condition, since in this work, the observations were considered in 2 seconds, and the estimation of fnmin depends only on the number of observations rather than the time interval between observations. For the real space-borne GNSS-R observations, the effects of temporal correlation for the observations should be carefully studied. Under Gaussian noise assumption of ionospheric delay, 80% of the values were less than 55, corresponding to a distance along the orbit track of 1100 km. In other words, the ionospheric delay was filtered with good effect on a 1000-km spatial scale. The peak value of fnmin was obtained when fnmin was 29, corresponding to a distance along the orbit trace of 600 km. This means that under experimental assumptions, the optimal spatial filter statistical results for ionospheric delay lie in a distance range from 400 to 1000 km, which is determined by the spatial distribution of the ionosphere. Varied noise backgrounds were tested for statistical analysis to exclude occasionality. 

In traditional altimetry technology, such as Jason series satellites and other altimetry satellites, the altimetry accuracy is relatively high at the centimeter level. For such high-precision altimetry, ionospheric delay correction was implemented, but there were still observation errors in the dual frequency calibration of ionospheric delay. To further improve the accuracy, the observation error needs to be eliminated, and smooth filtering is generally applied. The proposed spatial filtering distances of the Jason satellite were 100~150 km and 150~200 km, corresponding to 6:00–24:00 and 0–6:00 local time, respectively; that is, the spatial filtering reference distance of ionosphere delay in different local times is given. At present, there are no ionospheric delay observation data in space-borne GNSS-R satellites. Therefore, the simulation method was used to analyse the spatial filtering of ionospheric delay for space-borne GNSS-R altimetry. These experimental results can provide a useful reference for the spatial filtering method of ionospheric delay observation errors in GNSS-R altimetry and set the basis for follow-up research in related fields. More accuracy analysis will be conducted for the distribution of optimal spatial filtering points in different local times.

## 5. Conclusions

In this paper, a spatial filtering method of ionospheric delay observations in space-borne GNSS-R altimetry was studied. The ionospheric delay observations were computed with the GIM model provided by IGS. The spatial filtering analysis of these ionospheric delay observations was conducted with the absolute bias between the estimated value and the true value as the criterion to evaluate the filtering quality. The curves of absolute bias in relation to the bilateral filtering points fn  were verified to achieve the minimum absolute bias. The global distribution of fnmin was investigated and compared with the ionospheric correction derived from the GIM, and it showed that fnmin and ionospheric correction were negatively correlated. The optimal filtering points fnmin were evaluated with two methods. One method used a statistical analysis to find the maximum probability density at fnmin=20,28,32, corresponding to an altimetry accuracy of 10, 20, and 30 cm. The other method selected filtering points with a minimum value of the 99% quantile of $\sigma \left(fn\right)/\sigma_{0}$ to obtain a more stable filtering effect at fn = 10 corresponding to an altimetry accuracy of 10, 20, and 30 cm, with less computation assumption when fn = 10.
